# Organoid drug screening report for a non-small cell lung cancer patient with EGFR gene mutation negativity: A case report and review of the literature

**DOI:** 10.3389/fonc.2023.1109274

**Published:** 2023-02-16

**Authors:** Yuetian Pan, Hongshang Cui, Yongbin Song

**Affiliations:** ^1^ Medical Faculty of Ludwig-Maximilians-University of Munich, Ludwig-Maximilians-University, Munich, Bayern, Germany; ^2^ Department of Thoracic Surgery, Hebei General Hospital, Shijiazhuang, Hebei, China

**Keywords:** cancer, organoid, genetic analysis, lung cancer, case report

## Abstract

Patients with non-small cell lung cancer (NSCLC) who carry epidermal growth factor receptor (EGFR) mutations can benefit significantly from EGFR tyrosine kinase inhibitors (EGFR TKIs). However, it is unclear whether patients without EGFR mutations cannot benefit from these drugs. Patient-derived tumor organoids (PDOs) are reliable *in vitro* tumor models that can be used in drug screening. In this paper, we report an Asian female NSCLC patient without EGFR mutation. Her tumor biopsy specimen was used to establish PDOs. The treatment effect was significantly improved by anti-tumor therapy guided by organoid drug screening.

## Introduction

The 5-year survival rate of lung cancer is only 18% (1). Non-small cell lung cancer (NSCLC) accounts for 75%–80% of all lung cancers. Epidermal growth factor receptor (EGFR) is a tyrosine kinase receptor that activates cell proliferation pathways on the cell surface. Approximately 50% of Asian NSCLC patients harbor EGFR mutations (2, 3). Therefore, EGFR mutation detection has become a mandatory part of the management of Asian NSCLC patients (4, 5). Deletion-Exon19 (Del19) and exon 21 L858R substitution (L858R) account for 90% of EGFR mutations in NSCLC (6). Patients with EGFR mutations can benefit greatly from EGFR tyrosine kinase inhibitors (EGFR TKIs). However, due to the limitations of detection methods and the complexity of lung cancer, there are still many factors that will affect the accuracy of EGFR mutation detection. It is important to develop patient-specific drug prediction models for personalized anti-tumor therapy.

Patient-derived tumor organoids (PDOs) are three-dimensional models produced from a patient’s cancer tissue, combining genetic analysis with drug screening (7). PDOs can be used to simulate the occurrence and development of lung cancer, study the mechanism of drug resistance, and trace mutated genes (8). PDO can also be used *in vitro* for lung cancer drug screening, biomarker validation, and personalized anti-tumor drug treatment response prediction (9).

Here, we report an Asian female NSCLC patient without EGFR mutation. Anti-tumor therapy was administered based on drug screening of PDOs. The outcome of the patient after treatment was significantly improved. This case report provides a case basis for PDOs to become a clinical prediction model, helping in the development of precision medicine.

## Case presentation

This case involves a 66-year-old non-smoking Asian female NSCLC patient. She was admitted to the hospital for chest tightness, shortness of breath, intermittent cough for 1 year, and left chest pain for 1 month. The patient had no family history of lung tumors and had not previously received anti-tumor therapy. The patient’s vital signs were stable except for a few cardiac arrhythmias. Moreover, other hematological tests were in normal ranges except for tumor markers (carcinoembryonic antigen (CEA), 2.60 ng/ml; neuron-specific enolase (NSE), 18.80 ng/ml; CYFR21-1, 14.48 ng/ml). However, chest computed tomography (CT) showed a large space-occupying lesion in the upper lobe of the left lung with a maximum cross-sectional area of 7.3 cm × 5.5 cm. Moreover, there were multiple metastatic lymph nodes in the mediastinum and neck.

The patient could not receive surgical treatment due to the vast lesion and multiple lymph node metastases. Therefore, a tracheoscopic biopsy was performed immediately under flexible diagnostic bronchoscopy. The pathology report showed that the vast lesion was a hypofractionated adenocarcinoma classified as an epithelial tumor of NSCLC with high malignancy (No. 215758 from Hebei General Hospital Pathology Department).

Targeted therapy is a highly effective treatment option for Asian non-smoking female lung adenocarcinoma patients with an EGFR mutation. In this case, EGFR mutation detection was first performed at ShuWen Biology Laboratory. The pathology department provided formalin-fixed paraffin-embedded sections for the genetic testing. Regarding eight types of EGFR mutations, including Deletion-Exon19, L858R, and Insertion-Exon20, the test report suggested wild type with insensitivity to EGFR TKIs (such as gefitinib and erlotinib). The report (No. W0113008960) is shown in **Table 1**. The T790M mutation is the main reason for resistance to EGFR TKIs in patients with advanced NSCLC (8). Therefore, T790M mutation testing at Guangzhou KingMed Center was carried out. The center used the QX200 Droplet Digital PCR system with Human EGFR Gene T790M Mutation Detection Kit (S-ddPCR) to detect the T790M mutation. However, this report (No. QS20D-3953) also showed a lack of the T790M mutation. Because of the EGFR mutation negativity, the patient only received standard chemotherapy with a pemetrexed and carboplatin regimen. However, the patient experienced nausea and vomiting and could not eat during chemotherapy. Choosing an appropriate and effective treatment for her was a major dilemma.

**Table 1:** Human EGFR mutation detection report from ShuWen Biology Laboratory.

Project		Test method		Test instrument	
Detection of human EGFR gene mutation		ARMS		ABI7500	
Test result					
Project	CT value		Result		Reference range
Insertion-Exon20 mutation	Undetermined		Wild type		Wild type
G719X mutation	Undetermined		Wild type		Wild type
L858R mutation	Undetermined		Wild type		Wild type
L861Q mutation	Undetermined		Wild type		Wild type
S768I mutation	Undetermined		Wild type		Wild type
T790M mutation	Undetermined		Wild type		Wild type
Deletion-Exon19 mutation	Undetermined		Wild type		Wild type
Deletion-Exon21 mutation	Undetermined		Wild type		Wild type

The laboratory uses specific primers for highly accurate PCR amplification of mutant target sequences. A double-loop probe was used to detect amplification products. At the same time, the detection of rare mutations in sample DNA is achieved using a real-time fluorescent quantitative PCR platform to achieve high specificity and sensitivity for detecting mutations.

**Figure 1:**
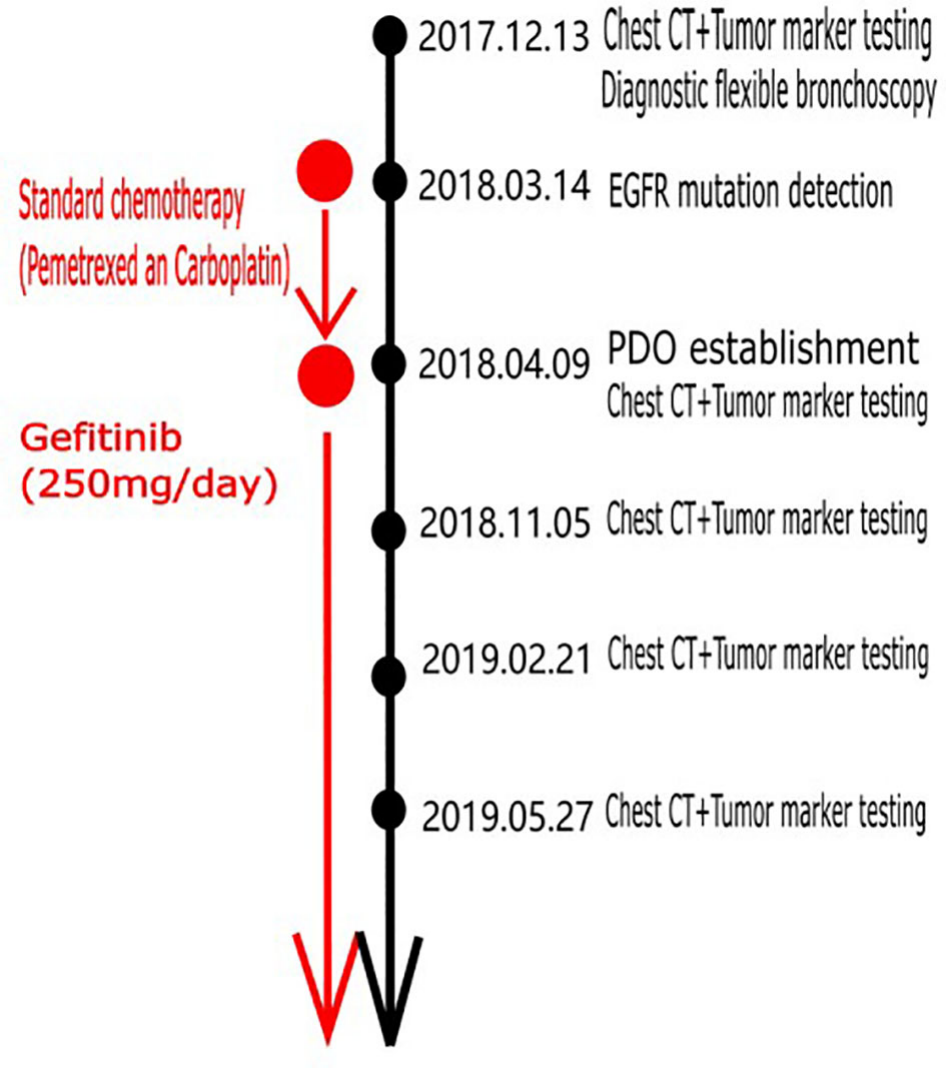
Timeline of the detection and therapeutic process.

A study involving 84 advanced lung cancer organoid models demonstrated that PDOs largely preserve somatic alterations in patients with advanced lung cancer, including the driving genes of tumors (10). Therefore, a CT-guided lung biopsy was performed, and we sent fresh tumor tissue samples to Beijing K2 Oncology Laboratory and successfully established a lung cancer PDO (OrganoidPro™ culture kit, K20-M-NSCLC). The PDO was treated with different concentrations and combinations of antineoplastic drugs. ATP quantification by CellTiter-Glo^®^ 3D Assay was used to detect tumor cell viability (CellTiter-Glo^®^ 3D Cell Viability Assay, Promega, Madison, WI, USA; G9681). Subsequently, a POLARstar Omega fully automated multifunctional device was used for the detection of enzyme markers. The results showed that carboplatin was ineffective at inhibiting the cancer cells, as was pemetrexed. However, gefitinib was 78% effective. Although carboplatin combined with pemetrexed was ineffective at inhibiting the cancer cells, gefitinib combined with carboplatin combined with pemetrexed was 78% effective. This report provides different information from the EGFR mutation report. The PDO drug screening report (No. KOLU-223) is shown in **Table 2**. Finally, standard chemotherapy was discontinued, and the patient was administered gefitinib for targeted therapy. The timeline of the diagnostic and therapeutic process is shown in **Figure 1**.

**Table 2:** The PDO drug screening result from K2 Oncology Laboratory.

Drug types	Drug	Organoid drug sensitivity result	Cancer cell inhibition rate
Chemotherapy	Carboplatin	Invalid	<10%
	Nedaplatin	Possibly valid	~30%
	Pemetrexed	Invalid	<10%
	5-Fluorouracil	Invalid	<10%
	Docetaxel	Invalid	<10%
Target therapy	Gefitinib	Valid	78%
	Dasatinib	Invalid	<10%
	Everolimus	Invalid	<10%
Combination therapy	Carboplatin+pemetrexed	Invalid	<10%
	Gefitinib+carboplatin+pemetrexed	Valid	78%
	Nedaplatin+pemetrexed	Possibly valid	~50%
	Gefitinib+nedaplatin+pemetrexed	Valid	75%

According to this organoid drug screening report, the patient was treated with gefitinib as monotherapy.

PDO, patient-derived tumor organoid.

**Figure 2:**
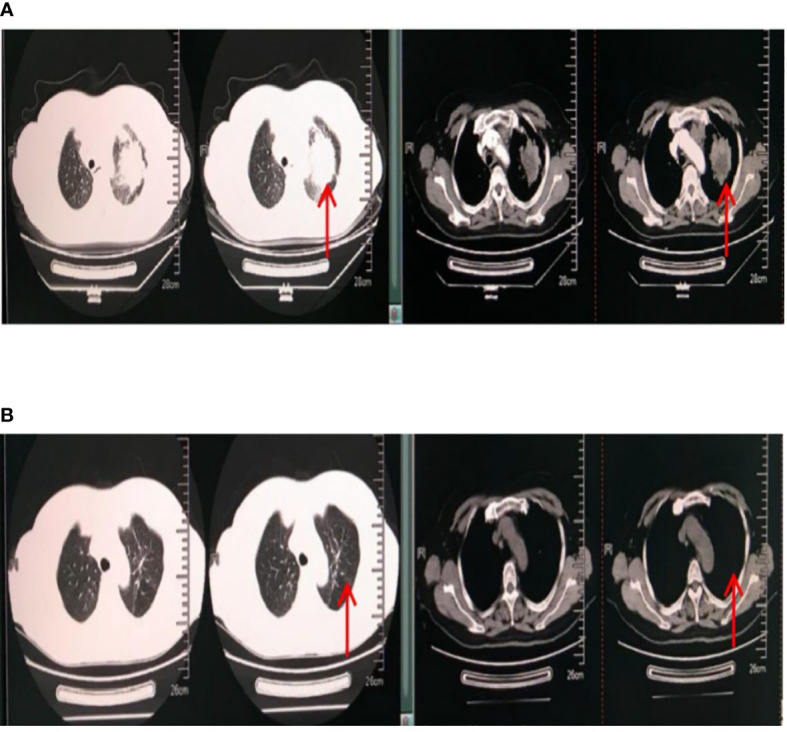
Chest CT results. The red arrow points to the lung tumor. **(A)** Chest CT results on 13 December 2017 (before treatment). The maximum cross-sectional area of the space-occupying lesion in the upper lobe of the left lung was 7.3 cm × 5.5 cm. Multiple metastatic lymph nodes were found in the mediastinum and neck. There was pleural effusion in the left chest. **(B)** Chest CT results on 27 May 2019 (after treatment). The maximum cross-sectional area of the space-occupying lesion was 9 mm × 4 mm. Multiple small lymph nodes were found in the mediastinum and neck.

The treatment of this patient was encouraging. The patient was followed up several times over 2 years. First, her vital signs were stable. Other symptoms, such as chest tightness, shortness of breath, and chest pain, were significantly alleviated. Only a mild cough remained. In addition, her tumor markers gradually decreased to the normal range (CEA, 1.59 ng/ml; NSE, 9.98 ng/ml; CYFR21-1, 1.64 ng/ml). Chest CT also showed a gradual reduction of the tumor to 9 mm × 4 mm. The patient’s living conditions improved significantly. The anti-tumor effect of the patient was evaluated as complete remission (CR). The chest CT results before and after treatment are shown in **Figure 2**.

## Discussion

With the advancement of molecular testing technologies, genetic testing has become a mandatory component in the management of NSCLC (4, 5). One study enrolling 1,482 patients from seven Asian countries and regions found that the overall EGFR mutation rate in Asian NSCLC patients was 51.4% (11). Therefore, EGFR has become the most common target for mutation testing.

The patient reported herein was diagnosed with pulmonary adenocarcinoma and was a non-smoking Asian woman. Her characteristics were fully consistent with those of EGFR mutation (12, 13). However, her EGFR genetic test results showed negativity for eight EGFR mutation types (including the classic mutation type Del19 in Asian patients), with EGFR TKI insensitivity. It is worth mentioning that the patient was only tested for eight types of EGFR mutation. Other mutational tests were not performed. We suggested that the patient proceeds with KRAS or ALK testing. Additionally, we suggested that the entire EGFR gene and the whole exon be sequenced to find rare mutations that activate the EGFR receptor. Unfortunately, the patient rejected these recommendations due to the high cost and long testing period. However, Deletion-Exon19, L858R, T790M, and Insertion-Exon20, the four most common mutation types in Asian women, were all negative (14). Thus, finding a more reliable drug sensitivity assay and providing patients with accurate treatment have become our greatest challenges.

PDOs have the advantage of saving time and cost. These models can be successfully established in 14 days (15). We successfully established a PDO for this patient with fresh tumor tissues from CT-guided lung biopsy and obtained a very interesting result from PDO drug screening assays. The combination of carboplatin, pemetrexed, and gefitinib did not increase cancer cell inhibition over gefitinib alone. This result indicated that the patient had undergone heavy physical burden of standard chemotherapy with little benefit. Based on the PDO drug screening result, the patient received gefitinib monotherapy.

With the help of the PDO drug screening result, patients can be treated appropriately and effectively. First, the physical and financial burden caused by chemotherapy can be avoided. Compared with chemotherapy, gefitinib only needs to be taken orally once daily, which greatly improves patient compliance and quality of life. Second, the treatment effect was very good in our case. The tumor was significantly reduced. The concentration of the detected tumor markers also decreased to the normal range. Her discomfort symptoms were also significantly relieved. Third, the whole treatment period was shorter. The accurate prediction of PDO greatly shortened the exploration period of effective treatment. In this regard, tumor patients will benefit greatly, especially those with advanced tumors (16). Finally, PDO drug screening can accurately predict multiple drugs rather than assess tumor response in the context of genomic profiling for mutations (17). For example, gefitinib is a first-generation targeted drug, whereas dasatinib is a second-generation targeted drug. Gefitinib’s cancer cell inhibition rate in PDO was 78%, significantly higher than that of dasatinib. This result shows that the advantage of accurate drug prediction by PDO over single-gene mutation prediction by genetic testing is clear.

However, there are still limitations in this case report. First, the patient was tested only for common EGFR mutations and did not receive genetic testing for other rare or different combinations of mutations. Performing all of these tests is difficult in the clinic. However, more comprehensive genetic testing data, or even high-throughput sequencing (HTS), is still necessary to explain negative EGFR mutation but effective TKI therapy. In 2015, Baik et al. reported a patient with a negative EGFR common mutation but effective EGFR TKI treatment (18). With next-generation sequencing (NGS), Baik found that EGFR exon 18–25 kinase domain duplication (EGFR-KDD) was the reason for the effective treatment of this patient. EGFR gene mutation is the most common mutation type in NSCLC patients, of which nearly 90% are Del19 and exon 21 L858R. However, there are still some mutation types with a small proportion that respond to EGFR TKI treatment. EGFR-KDD is a rare potential carcinogenic mutation, and its incidence in lung cancer is approximately 0.2% (19). Therefore, NGS can help patients obtain more accurate individualized treatment.

Second, Del19 and L858R were not detected in this case, but treatment with gefitinib was effective. In general, EGFR mutations are EGFR gene mutations that result in changes in the EGFR receptor on the cell membrane. Gefitinib is a small molecule receptor TKI that binds to the intracellular segment of the EGFR receptor to exert anti-tumor effects. There were no EGFR mutations detected in this case. However, gefitinib also inhibits insulin-like growth factor (IGF) and platelet-derived growth factor (PDGF), which are members of the tyrosine kinase receptor subfamily. It is unclear whether other tyrosine kinases and their signaling pathways can influence EGFR receptor mutations, affecting the efficacy of gefitinib therapy (20, 21).

## Literature review

In the literature, PDOs greatly preserve the histological and genetic characteristics of a primary tumor. First, Chen et al. (16) found >80% concordance between tumor and PDO samples for the top 20 NSCLC-related gene mutations. Second, PDOs were highly consistent with clinical treatment in terms of predicting the sensitivity of chemotherapeutic drugs (22). Similarly, in targeted therapy, PDO responses to targeted drugs correlated partially with the mutation profile, revealing similarities and differences between tumors and their corresponding PDOs. For example, Chen et al. (16) reported that PDOs show resistance to gefitinib but a therapeutic response to osimertinib. Matched clinical tumor tissues showed abnormal amplification of cMET, indicating the mechanism of gefitinib resistance. In addition, another PDO, with an insertion-exon 20 mutation, was resistant to gefitinib but exhibited a significant response to osimertinib and chemotherapy. Current genetic tests use genomic analysis to assess tumor response and can only predict the effect of drugs for this class of genetic spectrum mutations. It is difficult to predict the effectiveness of specific drugs (23). However, a drug screening assay was performed using the PDO for this patient, and the results showed that the PDO was resistant to gefitinib and sensitive to axitinib. Overall, there is a great advantage of PDO over genetic testing.

## Conclusion

Based on early experience with PDO in precision medicine, the advantages of PDO over genetic testing are clear. We recommend combining genetic testing with PDO drug screening assays, especially for patients with negative genetic testing results. Moreover, PDO can be used as an adjunctive approach to help clinicians identify the most beneficial drugs for their patients. Overall, we report an interesting case. Although NGS sequencing data are lacking, this case highlights the power of personalized treatment decisions guided by PDO drug screening and the value of combining different diagnostic tools (such as genetic testing and PDO drug screening). The finding will accelerate the development of precision medicine for lung cancer.

## Data availability statement

The original contributions presented in the study are included in the article/**Supplementary Material**. Further inquiries can be directed to the corresponding author.

## Ethics statement

This study was approved by the Medical Ethics Committee of Hebei Provincial People’s Hospital, No. (2018) Research Ethics No. (01). The patients/participants provided their written informed consent to participate in this study. Written informed consent was obtained from the individual(s) for the publication of any potentially identifiable images or data included in this article.

## Author contributions

YP, HC and YS performed the material preparation and data analysis. YP wrote the first draft of the manuscript. Also, YP and YS treated the patient. All authors contributed to the article and approved the submitted version.
