# Supplementary figures and images for: Organoid drug screening report for a non-small cell lung cancer patient with EGFR gene mutation negativity: A case report and review of the literature
Source: Front Oncol. 2023 Feb 16;13:1109274. doi: 10.3389/fonc.2023.1109274 (PMC9978590; doi:10.3389/fonc.2023.1109274)

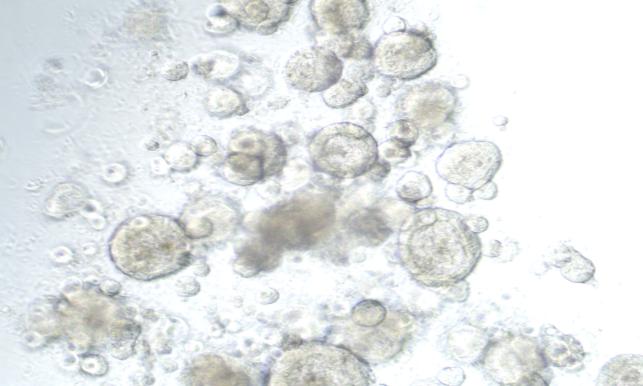

Supplement: Supplementary file 1 [file Image_1.jpeg]
