# Supplementary material for: Organoid drug screening report for a non-small cell lung cancer patient with EGFR gene mutation negativity: A case report and review of the literature
Source: Front Oncol. 2023 Feb 16;13:1109274. doi: 10.3389/fonc.2023.1109274 (PMC9978590; doi:10.3389/fonc.2023.1109274)
Supplement: Supplementary file 2 [file Table_1.doc]

| Project | Detection method | Result |
| --- | --- | --- |
| EGFR gene T79OM mutation (Digital PCR) KMCT | Digital PCR | Negative |
| Result interpretation | | |
| 1. No T790M mutation of EGFR gene was detected in this sample, please use this report in combination with other clinical information; | | |
| 2. This test only provides genetic information for clinical use. Please consult your clinician for specific medication. | | |

Supplementary Table 1. TM790 mutation detection report from Guangzhou KingMed Center Laboratory. TM790 mutation detection report from Guangzhou KingMed Center Laboratory.
