# Supplementary material for: Organoid drug screening report for a non-small cell lung cancer patient with EGFR gene mutation negativity: A case report and review of the literature
Source: Front Oncol. 2023 Feb 16;13:1109274. doi: 10.3389/fonc.2023.1109274 (PMC9978590; doi:10.3389/fonc.2023.1109274)
Supplement: Supplementary file 3 [file Table_2.doc]

| Date | Tumor Markers | | | |
| --- | --- | --- | --- | --- |
|  | CEA(ng/ml) | NSE(ng/ml) | CYFR21-1(ng/ml) | SCC(ng/ml) |
| 12.2017 | 2.60 | 18.80 | 14.48 | 1.554 |
| 11.2018 | 2.20 | 12.18 | 1.55 | 1.043 |
| 02.2019 | 1.59 | 9.98 | 1.64 | 1.131 |

Supplementary Table 2. Tumor marker results during treatment. As the targeted therapy proceeded, the patient's tumor markers decreased significantly. Among them, CEA, NSE and CYFR21-1 all decreased to normal levels.
